# Effects of housing stability and contemporary mortgage lending bias on breast cancer stage at diagnosis among older women in the United States

**DOI:** 10.1002/cam4.7397

**Published:** 2024-07-19

**Authors:** Nicole Rademacher, Yuhong Zhou, Emily L. McGinley, Purushottam W. Laud, Tina W. F. Yen, Sara Beltrán Ponce, Ann B. Nattinger, Kirsten M. M. Beyer

**Affiliations:** ^1^ Medical College of Wisconsin Milwaukee Wisconsin USA; ^2^ Institute for Health & Equity, Medical College of Wisconsin Milwaukee Wisconsin USA; ^3^ Center for Advancing Population Science, Medical College of Wisconsin Milwaukee Wisconsin USA; ^4^ MCW Cancer Center, Medical College of Wisconsin Milwaukee Wisconsin USA; ^5^ Division of Surgical Oncology, Department of Surgery Medical College of Wisconsin Milwaukee Wisconsin USA; ^6^ Department of Radiation Oncology Medical College of Wisconsin Milwaukee Wisconsin USA; ^7^ Department of Medicine Medical College of Wisconsin Milwaukee Wisconsin USA

**Keywords:** breast cancer, diagnosis stage, housing access, housing stability, mortgage lending bias

## Abstract

**Background:**

Interventions aimed at upstream factors contributing to late‐stage diagnoses could reduce disparities and improve breast cancer outcomes. This study examines the association between measures of housing stability and contemporary mortgage lending bias on breast cancer stage at diagnosis among older women in the United States.

**Methods:**

We studied 67,588 women aged 66–90 from the SEER‐Medicare linked database (2010–2015). The primary outcome was breast cancer stage at diagnosis. Multinomial regression models adjusted for individual and neighborhood socio‐economic factors were performed using a three‐category outcome (stage 0, early stage, and late stage). Key census tract‐level independent variables were residence in the same house as the previous year, owner‐occupied homes, and an index of contemporary mortgage lending bias.

**Results:**

In models adjusted for individual factors, higher levels of mortgage lending bias were associated with later stage diagnosis (RR = 1.10, 95% CI 1.02–1.20; RR = 1.31, 95% CI 1.16–1.49; RR = 1.41, 95% CI 1.24–1.60 for least to high, respectively). In models adjusted for individual and neighborhood socio‐economic factors, moderate and high levels of mortgage lending bias were associated with later stage diagnosis (RR = 1.16, 95% CI 1.02–1.33 for moderate and RR = 1.18, 95% CI 1.02–1.37 for high). Owner occupancy and tenure were not associated with later stage diagnosis in adjusted models.

**Conclusions:**

Contemporary mortgage lending bias demonstrated a significant gradient relationship with later stage at diagnosis of breast cancer. Policy interventions aimed at reducing place‐based mortgage disinvestment and its impacts on local resources and opportunities should be considered as part of an overall strategy to decrease late‐stage breast cancer diagnosis and improve prognosis.

## 1 BACKGROUND

Breast cancer is the second leading cause of cancer deaths in women in the United States, and women diagnosed with breast cancer at more advanced stages have poorer prognoses.^1^, ^2^ Black/African American women have increased risk of advanced stage breast cancer at diagnosis compared to White women.^3^, ^4^ The reasons for these disparities are not fully understood, but recent studies point to the influence of upstream social and environmental drivers. One such socio‐environmental factor that has received increasing attention is housing. The influence of housing on health is often considered in a multidimensional fashion, including concepts of housing access, quality, cost, and stability as well as both historical and contemporary structural barriers to securing housing, such as redlining and contemporary mortgage lending bias. Housing contributes to a myriad of health outcomes,^5^, ^6^ including cancer.^7^, ^8^, ^9^, ^10^, ^11^, ^12^, ^13^

The relationship between housing and health is measured by varying characteristics with several key themes, including access and stability. Housing stability is often described as housing tenure or occupant consistency.^6^, ^14^, ^15^, ^16^ Housing access can include access to both home ownership and rental units and may include aspects of bias in accessibility based on characteristics of the applicant or property location. One such bias in accessibility is contemporary mortgage lending bias. Contemporary mortgage lending bias is an economic investment measure defined as the modern practice of lenders denying mortgage loans based on property location. Historically, redlining was a practice where geographic areas deemed high default risk were graded as “undesirable” and marked in red ink on map allowing for segregationist mortgage lending, a form of systemic racism.^17^, ^18^, ^19^ Though laws such as the Fair Housing Act and Home Mortgage Disclosure Act (HMDA) have been enacted to stop such practices, racialized spatial patterns of mortgage investment persist.

Studies have incorporated measures of historical redlining and contemporary mortgage lending bias to understand the impact of these practices on cancer outcomes.^7^, ^8^, ^9^, ^10^, ^11^ A recent national study used a novel redlining index to demonstrate that contemporary mortgage lending bias is associated with poorer overall and breast cancer‐specific survival.^11^ The relative impacts of housing characteristics on breast cancer stage at diagnosis represents a gap in knowledge. The objective of this study is to examine the association between measures of housing stability and contemporary mortgage lending bias on breast cancer stage at diagnosis among older women living in the United States.

## 2 METHODS

### 2.1 Data sources and study cohort

Data were collected from the SEER (Surveillance, Epidemiology, and End Results)‐Medicare linked database that includes all incident breast cancer within SEER areas for individuals enrolled in Medicare. The cohort included in this study comprised women aged 66–90 years with first breast cancer diagnosis between 2010 and 2015 who reside in a Metropolitan Statistical Area (MSA) within a SEER area (*N* = 80,147). The population's scope was confined to individuals within an MSA, as the contemporary mortgage lending bias index was crafted to identify regions specifically within MSAs with properties less likely to secure mortgages. The cohort was restricted to those with a valid 2010 census tract for residence at diagnosis (*N* = 79,065) and with a valid value for the key predictors (*N* = 78,702). Additionally, the cohort was required to be enrolled in Medicare Parts A and B for 12 months prior to diagnosis to allow for calculation of comorbidity status and have documented stage to be included in the final study cohort (*N* = 67,588).

### 2.2 Outcome measures

The primary outcome measure was breast cancer stage at diagnosis (based on the SEER adjusted 6th edition American Joint Committee on Cancer (AJCC) TNM staging system).^20^ Stage was categorized as ductal carcinoma in situ (DCIS, stage 0), early stage (I–II), or late stage (III–IV).

### 2.3 Key predictors

The key independent variables, all measured at the census tract level, were percentage of population age 1+ years living in the same house as the previous year (tenure), percentage of occupied housing units that were occupant‐owned, and a previously described^7^ index of contemporary mortgage lending bias. The first two variables were obtained from the 2011–2015 American Community Survey 5‐year estimates. The mortgage lending bias index^7^ was estimated from HMDA data in 2007–2013 at the census tract level. It measures the odds ratio of denial of a mortgage application for properties in a local area compared to properties in the rest of the same MSA, controlling for the sex of the applicant and the loan amount to income ratio. The value of the measure ranges from zero to infinity. Values less than one indicate lower levels of denial of mortgage applications locally compared to the MSA as a whole. Values greater than one indicate higher levels of denial of mortgage applications, meaning local properties are less likely to secure mortgages. Mortgage lending bias values were grouped into four categories following published work using three break points (0.5, 1, and 2) to represent Least (0–0.5), Low (0.5–1), Moderate (1–2), and High (≥2) levels of mortgage lending bias.^11^ All variables were linked to cohort data based upon census tract of residence for the patient at diagnosis.

### 2.4 Covariates

Individual level factors considered in our adjusted models included age, race and ethnicity, dual enrollment status (Medicare and Medicaid), comorbidities, and marital status. Neighborhood level factors included income, percent of female householders, education, and employment. Patient age was derived from the Medicare enrollment file. SEER race and ethnicity variables were combined to create a single variable. Race was delineated as Hispanic, Non‐Hispanic Black, Non‐Hispanic White, Non‐Hispanic Other Race (Asian, Native American, Pacific Islander, and Unknown/Other). The Hispanic population was included as its own racial/ethnic group to maximize the number of individuals included in the Hispanic category. Medicaid and Medicare dual enrollment was included as a measure of individual level socioeconomic status, categorized as dual‐enrolled (Medicare plus Medicaid, a proxy for low‐income status) or Medicare only enrollment during the month of diagnosis. The number of comorbidities per individual (0, 1, or 2+) was determined using inpatient, outpatient, and carrier Medicare data for the 12 months prior to incident breast cancer diagnosis based on the Klabunde algorithm.^21^ Marital status was grouped as married, unmarried, or unknown. Income was defined as the median housing income, female householders was defined as the percentage of families with a female householder, education was defined as the percentage of the population 25 years and over with a high school education, and employment was defined as the percentage of unemployed civilians aged 16 years and over.

### 2.5 Analysis

Descriptive analyses were conducted to summarize the characteristics of the cohort. Each census‐derived housing variable was divided into tertiles to facilitate interpretation without breaking the predictor into too many variables, and mortgage lending bias was divided into Least (0–0.5), Low (0.5–1), Moderate (1–2), and High (≥2) categories. Multinomial logistic regression models were used given the three‐category stage outcome (DCIS, early, late). Unadjusted and adjusted models were estimated. Socioeconomic covariates were included in the adjusted models to account for the possibility that may impact breast cancer stage at diagnosis. In unadjusted models, only the key predictor was included and each of the three predictors was modeled separately. Covariates were then added and controlled for in each adjusted model, and the three predictors were again modeled separately. In all models, standard error was adjusted for MSA clusters. Additionally, the mortgage lending bias index was grouped by race/ethnicity to evaluate if race/ethnicity acted as a moderator in the relationship. Analysis was conducted using Stata statistical software.^22^

## 3 RESULTS

This study included 67,588 women aged ≥66 and enrolled in fee‐for‐service Medicare with incident breast cancer diagnosed between 2010 and 2015 (Table 1). Compared to women with DCIS or early‐stage invasive breast cancer, women diagnosed at later stages were more likely to have comorbidities, be single, or to be considered poor (based on Medicaid insurance status).

**TABLE 1:** Summary characteristics of incident female breast cancer cases in 2010–2015 within the SEER‐Medicare database by stage at diagnosis.

	DCIS (*n* = 11,323, % = 16.75)	Early stage (I–II) (*n* = 47,823, % = 70.76)	Late stage (III–IV) (*n* = 8442, % = 12.49%)	All stage (*n* = 67,588)
Age group, *N* (%)				
66–69 years	3304 (29.18)	11,587 (24.23)	1879 (22.26)	16,770 (24.81)
70–74 years	3450 (30.47)	13,389 (28.00)	2084 (24.69)	18,923 (28.00)
75–79 years	2392 (21.13)	10,345 (21.63)	1712 (20.28)	14,449 (21.38)
80–84 years	1495 (13.20)	7537 (15.76)	1473 (17.45)	10,505 (15.54)
85+ years	682 (6.02)	4965 (10.38)	1294 (15.33)	6941 (10.27)
Race, *N* (%)				
Non‐Hispanic White	8734 (77.14)	38,969 (81.49)	6601 (78.19)	54,304 (80.35)
Non‐Hispanic Black	1065 (9.41)	3587 (7.50)	949 (11.24)	5601 (8.29)
Hispanic	662 (5.85)	2671 (5.59)	520 (6.16)	3853 (5.70)
Non‐Hispanic other race	862 (7.61)	2596 (5.42)	372 (4.41)	3830 (5.67)
Asian	760 (6.71)	2210 (4.62)	310 (3.67)	3280 (4.85)
Native American	31 (0.27)	144 (0.30)	26 (0.32)	201 (0.30)
Pacific Islander	36 (0.32)	169 (0.35)	23 (0.27)	228 (0.34)
Other/unknown	35 (0.31)	73 (0.15)	13 (0.15)	121 (0.18)
Dual‐enrollee status (%)				
Dual‐enrolled^a^	1230 (10.87)	5826 (12.19)	1514 (17.94)	8570 (12.69)
Comorbidities, *N* (%)				
0	6125 (54.09)	24,714 (51.68)	4286 (50.77)	35,125 (51.97)
1	3018 (26.65)	12,711 (26.58)	2037 (24.13)	17,766 (26.29)
2+	2180 (19.25)	10,398 (21.74)	2119 (25.10)	14,697 (21.74)
Marital status, *N* (%)				
Single	5015 (44.29)	23,243 (48.60)	4887 (57.89)	33,145 (49.04)
Married	5604 (49.49)	22,328 (46.69)	3118 (36.93)	31,050 (45.94)
Unknown	704 (6.22)	2252 (4.71)	437 (5.18)	3393 (5.02)
Housing stability and access				
% owner‐occupied housing units^a^				
First tertile (0%–60.46%)	3622 (32.00)	15,504 (32.43)	3063 (36.29)	22,189 (32.84)
Second tertile (60.46%–79.76%)	3723 (32.89)	16,140 (33.76)	2740 (32.46)	22,603 (33.45)
Third tertile (79.76%–100%)	3975 (35.11)	16,169 (33.82)	2638 (31.25)	22,782 (33.71)
% in the same house 1 year ago^a^				
First tertile (0%–85.10%)	3603 (31.83)	15,922 (33.30)	2924 (34.64)	22,449 (33.22)
Second tertile (85.10%–90.56%)	3760 (33.21)	16,082 (33.63)	2749 (32.56)	22,591 (33.43)
Third tertile (90.56%–100%)	3958 (34.96)	15,812 (33.07)	2769 (32.80)	22,539 (33.35)
Mortgage lending bias index				
Least	1197 (10.57)	4757 (9.95)	737 (8.73)	6691 (9.90)
Low	6056 (53.48)	25,674 (53.69)	4125 (48.86)	35,855 (53.05)
Moderate	3131 (28.56)	13,657 (28.56)	2663 (31.54)	19,451 (28.78)
High	939 (8.29)	3735 (7.81)	917 (10.86)	5591 (8.27)

^a^ Some columns do not total 100% due to unknown values.

The cohort was predominately non‐Hispanic White (80.35%); 12% of this subgroup was diagnosed at late stage. The second and third largest racial and ethnic groups represented were non‐Hispanic Black (8.3%) and Hispanic women (5.7%); 17% and 13% of non‐Hispanic Black and Hispanic women were diagnosed at late stage, respectively. The mortgage lending bias index grouped by race/ethnicity is shown in Table 2. As the level of mortgage lending bias increased, the percentage of Non‐Hispanic White women decreased whereas the percentage of Hispanic and non‐Hispanic Black women increased.

**TABLE 2:** Contemporary mortgage lending bias level by race/ethnicity.

Percentage living in each level of mortgage lending bias by race/ethnicity	Least	Low	Moderate	High
Non‐Hispanic White	91.08	86.44	75.51	45.23
Non‐Hispanic Black	2.65	3.00	10.29	41.96
Hispanic	2.90	4.40	7.97	9.50
Non‐Hispanic other race	3.40	6.15	6.23	3.30

Both higher percentage of owner‐occupied units and higher percentage of persons living in the same home as 1 year ago were associated with decreased risk of late‐stage diagnosis in unadjusted analyses (second tertile RR 0.87 [0.79, 0.96] and third tertile RR 0.79 [0.72, 0.86] for owner‐occupied units; second tertile RR 0.90 [0.83, 0.98] and third tertile RR 0.86 [0.79, 0.94] for % in same unit; Table 3), but the relationships did not persist when the models were adjusted for individual or individual and neighborhood factors.

**TABLE 3:** Relative risk of the measures of housing stability and contemporary mortgage lending bias on stage at diagnosis. Risk measures are relative to the base outcome, DCIS. Standard error was adjusted for MSA clustering effects in all models.

Measure of housing stability and mortgage lending bias	A. Unadjusted		B. Adjusted for individual factors^a^		C. Adjusted for individual *and* SES neighborhood factors^a^	
	RR [95% CI]	*p* > |z|	RR [95% CI]	*p* > |z|	RR [95% CI]	*p* > |z|
*Early stage*						
% owner‐occupied units						
First tertile (0%–60.46%)	Base		Base		Base	
Second tertile (60.46%–79.76%)	1.01 [0.93, 1.10]	0.78	1.01 [0.92, 1.10]	0.82	1.03 [0.93, 1.14]	0.57
Third tertile (79.76%–100%)	0.95 [0.89, 1.01]	0.12	0.95 [0.89, 1.02]	0.14	1.14 [0.91, 1.08]	0.82
% in same unit as 1 year ago						
First tertile (0%–85.10%)	Base		Base		Base	
Second tertile (85.10%–90.56%)	0.97 [0.91, 1.03]	0.28	0.98 [0.92, 1.03]	0.40	0.98 [0.93, 1.04]	0.56
Third tertile (90.56%–100%)	0.90 [0.84, 0.97]	0.00	0.92 [0.85, 0.99]	0.03	0.93 [0.86, 1.01]	0.07
Mortgage lending bias index						
Least	Base		Base		Base	
Low	1.07 [1.00, 1.14]	0.05	1.08 [1.01, 1.15]	0.02	1.06 [0.99, 1.14]	0.08
Moderate	1.10 [1.03, 1.17]	0.01	1.12 [1.05, 1.20]	0.00	1.07 [1.00, 1.15]	0.04
High	1.00 [0.93, 1.07]	0.98	1.09 [1.01, 1.18]	0.02	1.03 [0.94, 1.12]	0.55
*Late stage*						
% owner‐occupied units						
First tertile (0%–60.46%)	Base		Base		Base	
Second tertile (60.46%–79.76%)	0.87 [0.79, 0.96]	0.01	0.96 [0.86, 1.07]	0.45	1.04 [0.92, 1.17]	0.57
Third tertile (79.76%–100%)	0.79 [0.72, 0.86]	0.00	0.91 [0.82, 1.01]	0.07	1.07 [0.94, 1.22]	0.30
% in same unit as 1 year ago						
First tertile (0%–85.10%)	Base		Base		Base	
Second tertile (85.10%–90.56%)	0.90 [0.83, 0.98]	0.01	0.95 [0.87, 1.03]	0.21	0.98 [0.91, 1.06]	0.66
Third tertile (90.56%–100%)	0.86 [0.79, 0.94]	0.00	0.94 [0.85, 1.03]	0.17	1.01 [0.91, 1.11]	0.90
Mortgage lending bias index						
Least	Base		Base		Base	
Low	1.11 [1.02, 1.20]	0.02	1.10 [1.02, 1.20]	0.02	1.07 [0.98, 1.17]	0.14
Moderate	1.38 [1.22, 1.56]	0.00	1.31 [1.16, 1.49]	0.00	1.16 [1.02, 1.33]	0.03
High	1.59 [1.42, 1.77]	0.00	1.41 [1.24, 1.60]	0.00	1.18 [1.02, 1.37]	0.03

^a^ Individual level factors: age, race and ethnicity, dual enrollment status, comorbidities, and marital status. Neighborhood level factors: income, percent of female householders, education, and employment.

Most notable in our analyses were the results of contemporary mortgage lending bias and stage at diagnosis (Table 3). For both the unadjusted and adjusted models, increasing degrees of mortgage lending bias (higher levels of mortgage denial) were significantly associated with increased risk of late‐stage diagnosis, demonstrating a clear gradient relationship. In the unadjusted model, as the mortgage lending bias index increased, the risk of late‐stage diagnosis increased (RR = 1.11 [1.02, 1.20], RR = 1.38 [1.22, 1.56], and RR = 1.59 [1.42, 1.77] for the Low, Moderate, and High groups, respectively). The adjusted models demonstrated the same pattern but with slightly attenuated relative risks (RR = 1.10 [1.02, 1.20], RR = 1.31 [1.16, 1.49], and RR = 1.41 [1.24, 1.60] for groups Low, Moderate, and High respectively when adjusted for individual factors; RR = 1.07 [0.98, 1.17], RR = 1.16 [1.02, 1.33], RR = 1.18 [1.02, 1.37] when adjusted for individual and neighborhood factors). Of note, once adjusted for individual and neighborhood factors, the RR for the low mortgage lending bias/late‐stage diagnosis group was no longer statistically significant.

## 4 DISCUSSION

In this study of 67,588 women with incident breast cancer, the key finding is that contemporary mortgage lending bias was significantly associated with later stage of breast cancer diagnosis among elderly women after adjusting for sociodemographic factors and comorbidities. A clear gradient was observed when comparing late‐stage diagnosis to stage 0 diagnosis. A higher percentage of patients living in the same house as the previous year and percentage of homes that were owner‐occupied were associated with decreased risk of late‐stage diagnosis in unadjusted models. However, these relationships were no longer statistically significant once adjusted for individual and neighborhood factors. One possible explanation is that the individual and neighborhood factors used in this study are mediators of the relationship between tenure and owner‐occupancy and control away the effect of the aforementioned predictors.

The explanations for the finding that contemporary mortgage lending bias was significantly associated with later stage of breast cancer diagnosis are not fully understood. One possible explanation is that areas with greater odds of mortgage denials receive generally less economic investment, limiting resources that support health‐promoting behaviors such as cancer screening. A lack of neighborhood resources could contribute to later stage breast cancer diagnosis. Additionally, it is important to acknowledge mortgage lending bias historically targeted certain racial and ethnic groups and continues to do so as Table 2 demonstrated.^19^ These groups have been marginalized throughout history and continue to have poorer health outcomes, including for breast cancer, but the relationship of mortgage lending bias and stage was not accounted for by race or other demographic and neighborhood variables in the multivariate models suggesting that race alone cannot account for these results.

Our study is subject to limitations. First, as the study uses the SEER‐Medicare database due to its many advantages, this study is limited to women aged 66–90 and findings may not be generalizable to younger populations; however the older age demographic does suffer the highest burden of deaths from breast cancer.^23^, ^24^ Although it required restricting the sample to ages 66+ rather than 65+, calculation of comorbidities were included in the study because breast cancer patients with chronic conditions could be more challenged in accessing services or have more frequent encounters with healthcare and thus be more likely to be screened. A second limitation is data on mammography screening rates was not available; therefore, it is possible that mammographic access is on the causal pathway between mortgage lending bias and later stage at diagnosis. Another limitation is that the data reflect where the women currently live rather than residential locations over their lifetimes; however, the literature demonstrates that residential mobility in the studied population is low.^25^ An additional consideration is that the time period for this study included the subprime mortgage crisis, which could have impacted estimates of the mortgage lending bias. Lastly, the measures of housing stability and access included in this study are not all‐encompassing. Studies of additional housing measures such as quality measures may further elucidate the role housing plays in stage at diagnosis of breast cancer and related cancer outcomes, and more research is needed.

Our findings align with recent studies demonstrating that housing access, specifically mortgage lending bias, plays a role in cancer outcomes. Similar to our study, one recent study^10^ found that historically redlined areas had higher risk for late‐stage diagnosis for multiple cancers, including breast cancer. However, these findings were limited to one state and considered historical redlining measures from the 1930s that are concentrated only on central city populations in a sample of US cities. Another recent study^11^ similarly found harmful effects of redlining on breast cancer outcomes. In this study, contemporary mortgage lending bias was associated with poorer breast cancer survival. Taken together, this study and the aforementioned studies suggest a critical role for both historical and contemporary mortgage lending bias in influencing cancer stage at diagnosis and outcomes. Upstream factors related to housing appear to be of importance in identifying target areas for intervention with the goal of improving cancer outcomes. Of note, while the prior studies examined numerous neighborhood factors, neither of these studies addressed additional characteristics of housing including tenure and owner occupancy, further highlighting the need for more research in this area.

## 5 CONCLUSION

In conclusion, among elderly women with breast cancer in the United States, women living in areas with higher levels of mortgage lending bias experience later stage breast cancer diagnoses than their counterparts living in areas with lower levels of mortgage lending bias. Contemporary mortgage lending bias is associated with late‐stage breast cancer at diagnosis. Future work should examine potential mediators of this relationship, which may offer specific targets for policy change. Further research should examine whether similar associations exist between contemporary mortgage lending bias and other health outcomes, including breast or other cancer‐specific outcomes. Additionally, our findings contribute to the growing body of literature regarding the negative effects of structural racism on health outcomes and serves as another call for strategic investments to eliminate structural racism and achieve health equity.

## AUTHOR CONTRIBUTIONS

**Nicole Rademacher:** Conceptualization (lead); data curation (lead); formal analysis (equal); investigation (equal); methodology (equal); project administration (lead); supervision (equal); validation (equal); visualization (equal); writing – original draft (lead); writing – review and editing (lead). **Yuhong Zhou:** Data curation (equal); formal analysis (equal); investigation (equal); methodology (equal); supervision (equal); validation (equal); visualization (equal); writing – review and editing (equal). **Emily L. McGinley:** Data curation (equal); formal analysis (equal); investigation (equal); methodology (equal); validation (equal); visualization (equal); writing – review and editing (equal). **Purushottam W. Laud:** Conceptualization (equal); formal analysis (equal); investigation (equal); methodology (equal); validation (equal); visualization (equal); writing – review and editing (equal). **Tina W. F. Yen:** Formal analysis (equal); investigation (equal); methodology (equal); validation (equal); visualization (equal); writing – review and editing (equal). **Sara Beltrán Ponce:** Investigation (equal); writing – review and editing (equal). **Ann B. Nattinger:** Formal analysis (equal); writing – review and editing (equal). **Kirsten M. M. Beyer:** Conceptualization (equal); data curation (equal); formal analysis (equal); funding acquisition (lead); investigation (equal); methodology (equal); project administration (equal); resources (lead); software (lead); supervision (equal); validation (equal); visualization (equal); writing – original draft (equal); writing – review and editing (equal).

## FUNDING INFORMATION

Supported by a grant from the National Cancer Institute (NIH/NCI R01CA214805). The collection of cancer incidence data used in this study was supported by the California Department of Public Health as part of the statewide cancer reporting program mandated by California Health and Safety Code Section 103885; the National Cancer Institute's SEER Program under contract HHSN261201000140C awarded to the Cancer Prevention Institute of California, contract HHSN261201000035C awarded to the University of Southern California, and contract HHSN261201000034C awarded to the Public Health Institute; and the Centers for Disease Control and Prevention's National Program of Cancer Registries, under agreement U58DP003862–01 awarded to the California Department of Public Health.

## CONFLICT OF INTEREST STATEMENT

None.

## IRB APPROVAL

This study was approved by the Institutional Review Board at the Medical College of Wisconsin.

## Data Availability

The data underlying this article were provided by SEER‐Medicare with permission under a data use agreement (DUA). Per the DUA, data cannot be shared. SEER‐Medicare data may be requested from SEER‐Medicare (https://healthcaredelivery.cancer.gov/seermedicare/obtain/). All other Geospatial data used (US Census and HMDA data) are publicly available.
